# Alleviating the adverse effects of salinity stress on *Salicornia persica* using sodium nitroprusside and potassium nitrate

**DOI:** 10.1186/s12870-023-04179-x

**Published:** 2023-03-29

**Authors:** Abbasali Moghaddam, Hamid Reza Larijani, Meysam Oveysi, Hamid Reza Tohidi Moghaddam, Mohammad Nasri

**Affiliations:** Department of Agronomy, Varamin-Pishva Branch, Islamic Azad University, Varamin, Iran

**Keywords:** SNP, NaCl, KNO_3_, Seed oil, Halophyte

## Abstract

**Background:**

Glasswort (*Salicornia persica*) is identified as a halophyte plant, which is one of the most tolerant plants to salt conditions. The seed oil of the plant contains about 33% oil. In the present study, the effects of sodium nitroprusside (SNP; 0, 0.1, 0.2, and 0.4 mM) and potassium nitrate (KNO_3_; 0, 0.5, and 1%) were evaluated on several characteristics of glasswort under salinity stress (0, 10, 20, and 40 dS/m).

**Results:**

morphological features, phenological traits, and yield parameters such as plant height, number of days to flowering, seed oil, biological yield, and seed yield significantly decreased in response to severe salt stress. However, the plants needed an optimal salinity concentration (20 dS/m NaCl) to obtain high amounts of seed oil and seed yield. The results also showed that a high level of salinity (40 dS/m NaCl) caused a decrease in plant oil and yield. In addition, by increasing the exogenous application of SNP and KNO_3_, the seed oil and seed yield increased.

**Conclusions:**

The application of SNP and KNO_3_ were effective in protecting *S. persica* plants from the deleterious effects of severe salt stress (40 dS/m NaCl), thereby restoring the activity of antioxidant enzymes, increasing the proline content, and maintaining cell membrane stability. It seems that both factors, i.e. SNP and KNO_3_, can be applied as mitigators of salt stress in plants.

## Background

Salt stress is one of the most destructive environmental factors that significantly limits the productivity of cultivated crops worldwide, especially in arid and semiarid regions [1]. The salinization of soils (1–3% per year) is an ongoing process and is expected to cause as much as 50% land loss by 2050 [2]. Salinity induces a multitude of responses in plants, including molecular, biochemical, physiological, and morphological changes that lead to a decrease in the yield and quality of crops [3, 4].

One of the most important strategies of agriculture in areas with abiotic stress, such as drought and salinity, is the cultivation of salinity-resistant species or cultivars [5]. In saline soils, plants that are salt–resistant can produce reasonable amounts of yield. Halophytes are characterized by different physiological, morphological, and biochemical mechanisms, and can grow in salt-affected areas [6].

*Salicornia persica* is identified as a native Iranian halophyte [7]. Among plant species, it is one of the most tolerant to salt conditions. Specifically, it is classified among 1560 halophytes and inhabits salty areas [8]. The glasswort plant is dwarf and is categorized as a C3 annual species, leafless with succulent stems. The glasswort needs an optimal level of salinity (170–200 mol m^− 3^ NaCl) for growth and development and, accordingly, has adapted to seawater (500 mM NaCl) in going through the stages of its lifecycle [9]. The dwarf glasswort is a unique vegetable introduced to Asia, Europe, and the Americas, with fresh produce that has entered markets successfully. Its succulent young shoots are regarded as ‘Sea asparagus’ or ‘Samphire’ in gourmet kitchens for their high nutritional value and salty taste. Also, glassworts can be used as animal feed, biodiesel, and in aviation biofuel production [10]. The seed oil of the plant is similar to safflower oil, i.e. the seeds contain about 33% oil and rich in linoleic acid (70%) [11]. The cultivation of seawater-irrigated glasswort can lead to a good potential in oil production.

Another main strategy to alleviate salinity stress in crop plants is the exogenous supplementation of various compounds such as potassium nitrate (KNO_3_) and sodium nitroprusside (SNP) [12, 13].

Potassium is a mineral nutrient that plays a key role in increasing plant tolerance to saline conditions [14]. It is involved in vital processes in plants such as the regulation of stomatal closure, photosynthesis, activation of enzymes, the regulation of Na^+^ uptake, the accumulation of carbohydrates, and the translocation of the same from source to sink in salt-stress conditions [15]. The availability of potassium in plants is strongly affected by salinity. Similarly, the increase in potassium content is one of the major mechanisms which mitigates the adverse effects of salt stress by preventing cellular damage. Sodium nitroprusside is a nitric oxide (NO) donor compound that plays an important role in plants under normal and stressful conditions. NO regulates many growth-related and developmental processes, including root growth, leaf extension, closure of stomata, maintenance of water status, increase in photosynthetic capacity, xylogenesis, flowering, aging, and cell-mediated death [16]. On the other hand, the application of SNP reportedly facilitated wound healing and reduced disease severity in different plants. It also increased plant tolerance to abiotic stresses such as salt, cold, heat, and drought. More explicitly, the external application of SNP under saline conditions enhanced the performance of many plants such as lentils, tomatoes, lettuce, maize, soybean, chickpea, rice, cotton, and wheat. Exogenously applied SNP improved phytopharmaceutical production and essential oil yield in *Origanum majorana* and *Thymus serpyllum* [17].

Climate change and an excessive use of underground water have worsened the condition of salinity in soil and water resources for agricultural lands in Iran [18]. In most of these areas, the cultivation of plants is becoming less possible because of susceptibility to salinity. Therefore, introducing salinity-tolerant plants is strongly advised for salt-affected areas. Therefore, the present study aimed to evaluate the effects of different levels of salinity on several characteristics of *S. persica*. In response to salinity, KNO_3_ and SNP treatments were applied exogenously to reduce the impact of salinity stress on plant characteristics.

## Materials and methods

### Plant materials and growth conditions

**Fig. 1:**
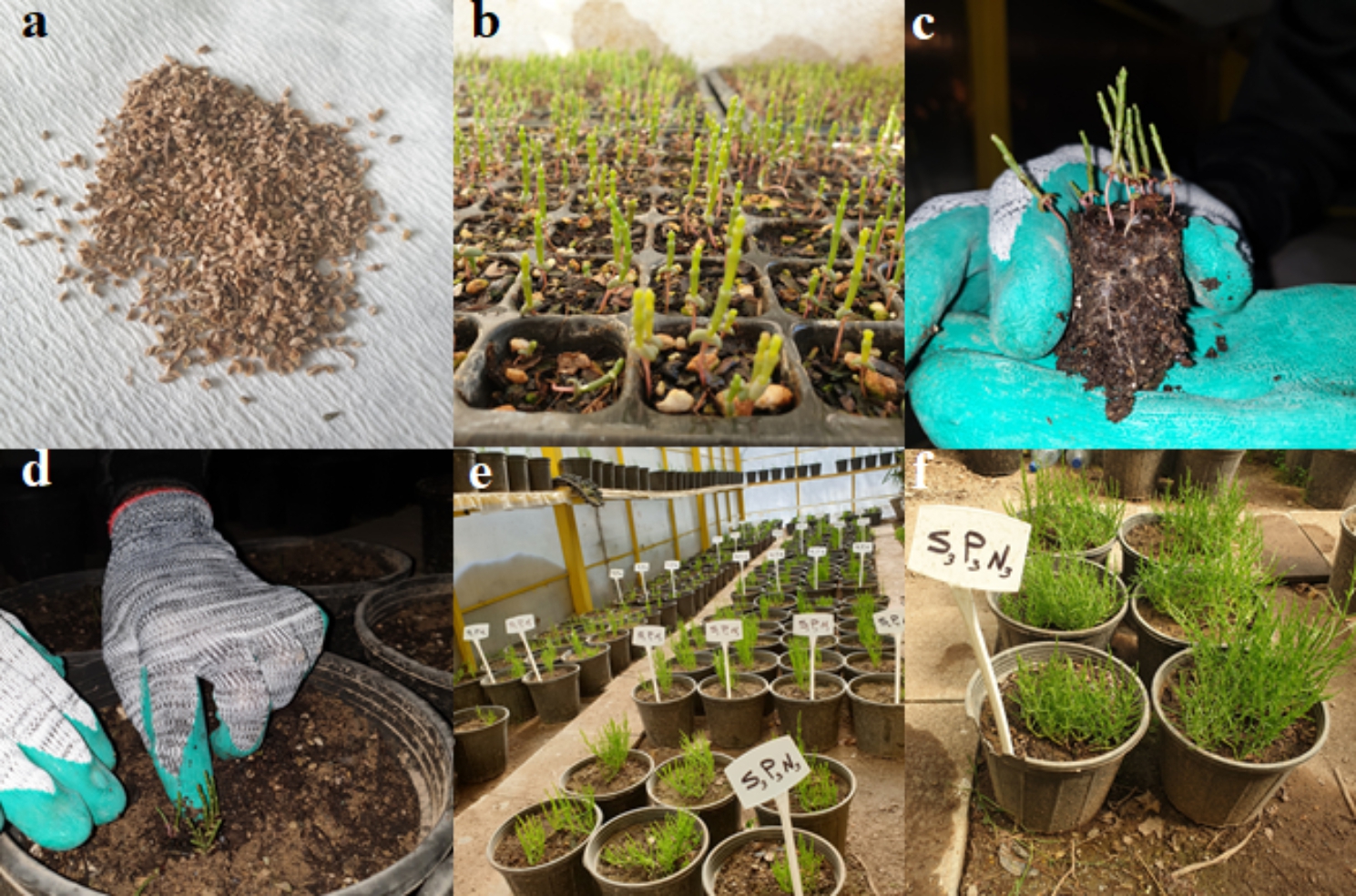
The stages of the experiment. (**a**) The plant seeds; (**b**) Seedlings growing; (**c**) Seedling ready for transplanted; (**d**) A seedling transplanted to a pod; (**e**) Plants growing; and (**f**) Plants at the end of experiment

In the present study, plant seeds were collected from inland salt marshes in Fars province, Iran. The seeds were surface sterilized in 10% (v/v) sodium hypochlorite solution, containing 0.1 ml of Tween 20 for 15 min. Then, they were washed three times with sterile distilled water. The seeds were planted in pots (37 cm deep and 25 cm in diameter) (Fig. 1). The pots were filled with sterilized loam soil (40% silt, 40% sand, and 20% clay) (Table 1) which had been sterilized in an oven at 180 °C for 8 h. The pots were placed in a greenhouse, with a 13-h photoperiod, relative humidity of 60 to 70%, a photosynthetic photon flux density of 550 µmol.m^–2^.s^–1^, and a temperature of 23 °C. For the proper establishment of plants, initial irrigations (for 3 months) were performed with tap water (0 dS/m of sodium chloride). The irrigation was carried out according to phenological needs and plant water requirements. The pots were watered to maintain soil moisture at field capacity. The plants were treated with four salt levels, i.e. 0, 10, 20, and 40 dS/m of sodium chloride. The salt stress was applied from the time of plant establishment (after three months of seed germination) to the time of harvest. Potassium nitrate (0, 0.5, and 1%) and sodium nitroprusside (0, 0.1, 0.2, and 0.4 mM) were separately applied as a foliar spray during the first three weeks of application of salinity stress. Only the salinity treatments were continuously applied until harvest. The operator ceased to apply potassium nitrate and sodium nitroprusside after three weeks, following plant establishment. This research was performed under greenhouse conditions, with 14 h light/10 h dark at 24–28 °C during the day, and 20–24 °C at night. The relative humidity was consistently maintained at 55%. Also, in the present study, all methods were performed in accordance with the relevant guidelines and regulations.

**Table 1:** The results of soil physical and chemical properties

EC (dS m^− 1^)	pH	Total neutralizing value	Organic carbon	P	K	N	Clay	Silt	Sand
		(%)		(mg kg^− 1^)			%		
1.18	7.79	11.4	2.3	5.88	279	0.05	20	40	40

### Plant height and number of days to flowering

The plant height was calculated as the linear distance between the base of the plant to the top of the last leaf at the end of the growing season. Measurements were made with an accuracy of 1 mm when the leaves began to change color. The number of days until flowering was measured one week after germination until flowering began.

### Chlorophyll content

One gram of crushed leaf sample was mixed with 5 mL of dimethyl sulfoxide (DMSO), and tubes were placed in a water bath (65 ^O^C) for four hours. Finally, 1 ml of supernatant was used for spectrophotometer (Spectronic Instruments, Rochester, NY) analysis at wavelengths of 645 and 663 nm [19, 20]. The content of chlorophyll a, b, and total chlorophyll were estimated based on formula 1:

(Chlorophyll a (mg/g) = (12.7 x A663) - (2.69 x A645))

(Chlorophyll b (mg/g) = (22.9 x A645) - (4.68 x A663))

Total chlorophyll (mg/g) = (20.2 x A645) + (8.02 x A663) (1).

### Sodium (Na^+^) and chlorine (Cl^−^)

The leaves were washed and then dried in an oven. The dried leaves were ground. After 48 h of extraction with 1 N HCl, the samples were analyzed by a flame photometer (Shimadzu CIM-1OlA) [21]. After drying, diluted water was added and samples were filtered twice. Finally, concentrations of chlorine were determined by an ion meter (Methrom- Switzerland). Concentrations of Na^+^ and Cl^−^ were expressed as mg/g dry weight.

### Biological yield and the thousand-grain weight

After harvest time, the plants were dried in an oven at 70 °C, and then the weight of the dried plants was calculated by a digital scale (A&D, Japan). Also, 1000 seeds of glasswort plants were collected and weighed.

### Seed oil and yield

The seeds were subjected to different treatments. They were dried and ground to prepare for the oil extraction process. After grinding, total oil was extracted using hexane in a soxhlet extractor by a continuous series of cycles of boiling and condensation of the solvent for 4 h [11]. Also, the seed yield was measured as grams per plant (g/plant).

### Antioxidant enzymes

The extraction of antioxidant enzymes was carried out by the method of Esfandiari et al. [22]. For catalase (CAT) and superoxide dismutase (SOD) extraction, leaf samples (0.5 g) were ground in chilled 0.1 M phosphate buffer solution (pH 7.5), and then the extract was centrifuged for 20 min at 8000 rpm (4 ^o^C). Finally, the supernatant was collected. The activity of SOD was assayed according to the method of Kumar et al. [23] by measuring nitroblue tetrazolium (NBT) suppression in photo-reduction. The extract included 18.75 µM NBT, 5 µM riboflavin, 25 µM EDTA, and 32 m ML-methionine in 250 ml distilled water. The reaction mixture contained enzyme extract (0.025 ml), d.H2O (0.25 ml), and 2.75 ml of the reaction solution. The SOD reaction was performed at 25 °C for 20 min under a light intensity of 4000 flux. The absorbance was observed at 560 nm with a spectrophotometer (Lambda 25 UV/VIS). The activity of CST was assayed according to the method of Kumar et al. [23]. The solution mixture included enzyme extract (0.1 ml), sodium phosphate buffer solution (50 mM; pH 7.0), H_2_O_2_ (300 mM), and deionized water (0.1 ml). Wavelengths were measured at 240 nm using a spectrophotometer (Lambda 25 UV/VIS).

### Malondialdehyde (MDA)

The MDA content was estimated by measuring the malondialdehyde content according to Esfandiari et al. [22]. Plant samples (500 mg) were homogenized in 2.5 ml of 5% trichloroacetic acid (TCA) and the aliquot was added with thiobarbituric acid (TBA) and 1.5 ml of crude enzyme extract. The solution was heated for 30 min to reach 95 °C and then was cooled down. The mixture was centrifuged for 10 min at 10,000 g. The absorbance was recorded at 532 nm by deducting the non-specific absorption at 600 nm.

### Proline content

The proline content was measured according to the method of Bates et al. [24] as follows. Leaf samples (0.5 g) were homogenized in 3.0% sulphosalicylic acid and then the homogenate was centrifuged at 1000 rpm for 10 min. Acid ninhydrin reagent (2 mL) and glacial acetic acid (2 mL) were added to the supernatant (1 mL). The mixture was placed at 100 °C for 1 h and then cooled suddenly in an ice bath. After cooling, toluene (4 mL) was added to the solution mixture and vortexed. The upper layer was transferred to a new test tube. Wavelengths were observed at 520 nm using a spectrophotometer (Lambda 25 UV/VIS).

### Statistical analysis and experimental design

The experiment was designed as a factorial experiment in a completely randomized design, with three factors including four concentrations of salt stress(0, 10, 20, and 40 dS/m) and three levels of SNP (0, 0.1, 0.2, and 0.4 mM), and KNO_3_ (0, 0.5, and 1%) with six biological replications. Data were tested by analysis of variance in SAS 9.1 (SAS Institute Inc., Cary, NC). Differences between the mean values were evaluated using Duncan’s test at a 5% probability level. The heat map correlation analysis (based on Pearson’s correlation coefficient) was generated as a colored heat map using MetaboAnalyst [25].

## Results and discussion

### ANOVA followed by post hoc LSD test

According to the results of ANOVA, there were significant differences (P < 0.01) among the effects of salt stress, nitrate potassium, SNP, and their interactions, which led to changes in plant height, days to flowering, biological yield, thousand-grain weight, seed oil, chlorophyll a, b, and total chlorophyll, concentrations of Na^+^ and Cl^−^, enzymatic antioxidants (SOD and CAT), malondialdehyde, and proline (Table 2). According to Wickens and Keppel [26], when the tripartite interaction was significant, the other effects were not considered significant.

**Table 2:** Analysis of variance (mean squares) for traits measured in *S. persica*

Source	df	PH	DF	BY	TW	SO	Cla	Clb	TCl	Na	Cl	SOD	CAT	MDA	Proline	SY
NaCl	3	2561**	10,887**	95.9**	0.42**	652**	134**	724**	264**	6512**	6136**	67.8**	24.3**	545**	0.02**	11.68**
SNP	3	109**	1453**	2.92**	0.12**	25.6**	88.5**	203**	469**	207**	250**	5.25**	5.24**	47.5**	0.005**	0.49**
KNO3	2	103**	1429**	7.1**	0.02**	2.85**	11.9**	61.2**	35.9**	41.6**	50.6**	0.57**	0.29 ^ns^	4.10**	0.001**	0.07**
NaCl×SNP	9	64.6**	364^ns^	0.53**	0.02**	10.2**	9**	12.6**	18.3**	14.5**	12.5**	1.15**	0.47**	5.46**	0.001**	0.05**
NaCl× KNO_3_	6	18.6 ^ns^	170^ns^	0.16 ^ns^	0.009**	0.53 ^ns^	0.98 ^ns^	4.79**	1.64**	9.21**	6.00 ^ns^	0.11**	0.12 ^ns^	0.42 ^ns^	0.001**	0.02*
KNO_3_× SNP	6	4.54**	98.3^ns^	0.21**	0.000 ^ns^	0.23 ^ns^	0.78 ^ns^	7.79**	2.63**	4.59**	10.4**	0.04 ^ns^	0.20 ^ns^	0.41 ^ns^	0.0001*	0.009 ^ns^
KNO_3_× SNP× NaCl	18	23.7**	148^ns^	0.44**	0.003**	0.63**	3.40^**^	1.82 ^ns^	2.59**	4.61**	6.21*	0.08**	0.12 ^ns^	0.61 ^ns^	0.0001*	0.013*
Error	240	9.22	239	0.07	0.0007	0.36	0.50	6.24**	0.67	2.04	3.14	0.02	0.10	0.54	2.04	0.007
CV (%)		9.67	8.44	14.05	6.92	3.46	9.25	1.21	8.03	6.1	8.54	5.35	9.60	8.51	3.48	10.29

PH: plant height, DF: the number of day until flowering, BY: biological yield, TW: thousand-grain weight, SO: seed oil, Ca: chlorophyll a, Clb: chlorophyll b, TCl: total chlorophyll, Na: concentration of Na^+^, Cl: concentration of Cl^−^, MDA: Malondialdehyde, SY: seed yield. ns = not significant; *, ** = significant at 5% and 1% level, respectively

### Plant height

Plant height is usually an important quantitative trait that can influence yield [27]. Salt stress is a major problem that strongly affects most of the morphological traits, including plant height. In response to salt stress, plant height usually decreases as a consequence of the osmotic extraction of intracellular water. According to the results, by increasing salinity levels from 0 to 20 dS/m, plant height increased. By 40 dS/m salinity, however, the plant height decreased (Table 3).

**Table 3:** Effects of salinity, SNP, and KNO_3_ on some characters of *persica*

	DF	PH (cm)	BY (g)	TW (g)	SO (%)	Cla (mg g^− 1^ FW)	Clb (mg g^− 1^ FW)	TCl (mg g^− 1^ FW)
Salinity (dS/m)								
0	195 ± 2.54^*^ a	24.05 ± 1.69 d	0.67 ± 0.12 d	0.34 ± 0.02 c	13.13 ± 0.57 d	6.62 ± 0.69 c	16.2 ± 1.24 b	9.84 ± 1.88 c
10	192 ± 3.22 a	35.17 ± 3.94 b	1.48 ± 0.24 c	0.42 ± 0.04 b	19 ± 0.95 b	9.26 ± 1.99 a	16.16 ± 2.15 a	12.5 ± 3.49 a
20	178 ± 2.12 b	37.08 ± 0.26 a	3.31 ± 0.17 a	0.49 ± 0.09 a	20.2 ± 1.57 a	8.36 ± 1.16 b	15.4 ± 2.82 c	10.7 ± 1.95 b
40	168 ± 1.87 c	30.04 ± 4.2 c	2.47 ± 0.33 b	0.33 ± 0.04 d	17.2 ± 0.41 c	6.44 ± 1.14 c	9.86 ± 1.96 d	7.88 ± 2.56 d
SNP (Mm)								
0	180 ± 2.38 b	29.93 + 0.9 b	1.75 + 0.23 d	0.34 + 0.05 d	16.62 + 0.24 d	6.31 + 0.12 d	12.6 + 0.26 d	22.7 + 1.4 a
0.1	180 ± 2.08 b	30.88 + 0.63 b	1.91 + 0.14 c	0.38 + 0.01 c	17.48 + 0.33 c	7.45 + 0.16 c	13.7 + 0.37 c	21.5 + 0.22 b
0.2	189 ± 2.44 a	33.1 + 0.73 a	2.05 + 0.07 b	0.42 + 0.06 b	17.74 + 0.34 b	7.96 + 0.17 b	15.41 + 0.21 b	20.2 + 1.24 c
0.4	185 ± 1.14 a	33.08 + 0.67 a	2.22 + 0.11 a	0.44 + 0.02 a	18 + 0.38 a	8.98 + 0.24 a	16.42 + 0.34 a	18.4 + 1.3 d
KNO3 (%)								
0	179 ± 3.62 b	30.1 ± 0.6 b	1.69 ± 0.11 c	0.38 ± 0.03 c	17.28 ± 0.28 b	7.33 ± 0.18 c	13.75 ± 0.26 c	9.85 ± 0.27 c
0.5	184 ± 2.51 a	31.96 ± 0.23 a	2.04 ± 0.06 b	0.4 ± 0.02 b	17.48 ± 0.29 a	7.66 ± 0.17 b	14.58 ± 0.13 b	10.32 ± 0.32 b
1	187 ± 2.02 a	32 ± 0.67 a	2.23 ± 0.03 a	0.42 ± 0.04 a	17.62 ± 0.29 a	8.04 ± 0.19 a	15.34 ± 0.30 a	10.8 ± 0.34 a
	Na (mg g^− 1^ DW)	Cl (mg g^− 1^ DW)	SOD (EU mg^− 1^ protein)	CAT (EU mg^− 1^ protein)	MDA (umol/g FW)	Proline (mg/g FW)	SY (g/plant)	-
Salinity (dS/m)								
0	11.9 ± 1.9 d	9.62 ± 1.42d	1.68 ± 0.09 d	2.73 ± 0.32 d	6.04 ± 0.7 d	0.17 ± 0.01 d	0.31 d	-
10	19.2 ± 2.55 c	16.81 ± 2.34 c	3.25 ± 0.28 b	3.65 ± 0.34 b	6.85 ± 1.18 c	0.20 ± 0.01 c	1 b	-
20	30.6 ± 1.63 b	26.85 ± 2.09 b	3.97 ± 0.58 a	4.08 ± 0.46 a	9.9 ± 1.25 b	0.22 ± 0.02 b	1.22 a	-
40	31.8 ± 2.83 a	29.83 ± 3.87 a	2.63 ± 0.26 c	3.2 ± 0.52 c	11.9 ± 1.17 a	0.25 ± 0.03 a	0.62 c	-
SNP (Mm)								
0	25.3 + 1.08 a	22.78 + 1.03 a	2.61 + 0.08 d	3.13 + 0.17 d	9.67 + 0.34 a	0.22 + 0.02 a	0.7 d	-
0.1	24.1 + 0.98 b	21.56 + 0.38 b	2.75 + 0.09 c	3.32 + 0.07 c	9.04 + 0.31 b	0.21 + 0.01 b	0.76 c	-
0.2	23.07 + 0.96 c	20.26 + 0.92 c	2.92 + 0.11 b	3.46 + 0.12 b	8.2 + 0.28 c	0.21 + 0.02 b	0.82 b	-
0.4	21.31 + 1.46 d	18.43 + 0.66 d	3.24 + 0.13 a	3.77 + 0.08 a	7.88 + 0.26 d	0.19 + 0.01 c	0.89 a	-
KNO3 (%)								
0	24.4 ± 0.67 a	21.57 ± 0.89 a	2.81 ± 0.04 c	3.36 ± 0.02 b	8.94 ± 0.18 a	0.23 ± 0.01 a	0.79 c	-
0.5	23.36 ± 0.85 b	20.53 ± 0.73 b	2.88 ± 0.07 b	3.43 ± 0.06 ab	8.6 ± 0.21 b	0.21 ± 0.01 b	0.82 b	-
1	22.84 ± 0.71 c	20.18 ± 0.77 b	2.96 ± 0.03 a	3.47 ± 0.11 a	8.56 ± 0.11 c	0.21 ± 0.02 b	0.89 a	-

^*^: values are mean ± standard error (SE); DF: the number of day until flowering, PH: plant height, BY: biological yield, TW: thousand-grain weight, SO: seed oil, Cla: chlorophyll a, Clb: chlorophyll b, TCl: total chlorophyll, Na: concentration of Na+, Cl: concentration of Cl-, MDA: Malondialdehyde. Different letters indicate significant differences among means using LSD test at P = 0.05

Our results showed that the application of potassium nitrate and SNP mitigated the destructive effects of sodium chloride on plant height (Table 4).

**Table 4:** The effects of tripartite interaction of NaCl, Sodium nitroprusside, and potassium nitrate on some characters of *S. persica*

S	P	K	PH	CB (mg g^− 1^ FW)	CT (mg g^− 1^ FW)	BY (g)	TW (g)	Na (mg g^− 1^ DW)	Cl (mg g^− 1^ DW)	SO (%)	SOD (EU mg^− 1^ protein)	Proline (mg/g FW)	Seed yield (g/plant)
0	0	0	21 ± 0.4	15.16 ± 0.3	7.01 ± 0.34	0.35 ± 0.02	0.35 ± 0.01	11.57 ± 1.3	10.77 ± 0.2	13.3 ± 0.2	1.66 ± 0.12	0.17 ± 0.01	0.27 ± 0.05
		0.1	21.5 ± 0.4	15.06 ± 0.5	7.83 ± 0.32	0.38 ± 0.02	0.32 ± 0.01	13.17 ± 0.3	10.59 ± 0.3	13.3 ± 0.2	1.62 ± 0.14	0.17 ± 0.02	0.28 ± 0.03
		0.2	22.33 ± 0.3	15.93 ± 0.3	7.77 ± 0.25	0.39 ± 0.03	0.35 ± 0.01	13.28 ± 0.8	11.07 ± 0.3	13.42 ± 0.3	1.56 ± 0.23	0.2 ± 0.03	0.28 ± 0.02
	0.1	0	23.17 ± 0.5	15.82 ± 0.3	8.86 ± 0.33	0.43 ± 0.02	0.35 ± 0.02	12.02 ± 1.2	10.78 ± 0.3	13.34 ± 0.4	1.58 ± 0.34	0.17 ± 0.01	0.34 ± 0.04
		0.1	24 ± 0.2	15.35 ± 0.3	8.92 ± 0.18	0.41 ± 0.03	0.35 ± 0.02	13.07 ± 0.8	10.47 ± 0.2	13.18 ± 0.4	1.68 ± 0.21	0.19 ± 0.02	0.31 ± 0.03
		0.2	24.83 ± 0.3	15.76 ± 0.4	9.63 ± 0.36	0.48 ± 0.01	0.34 ± 0.01	12.15 ± 0.3	9.73 ± 0.47	13.25 ± 0.2	1.71 ± 0.03	0.2 ± 0.01	0.34 ± 0.02
	0.2	0	24.17 ± 0.6	16.81 ± 0.4	10.42 ± 0.3	0.48 ± 0.02	0.34 ± 0.02	13.12 ± 0.9	9.52 ± 0.46	13.22 ± 0.2	1.64 ± 0.33	0.19 ± 0.03	0.34 ± 0.03
		0.1	24.33 ± 0.5	16.24 ± 0.5	10.32 ± 0.5	0.82 ± 0.13	0.34 ± 0.01	11.92 ± 0.3	10.27 ± 0.4	13.5 ± 0.07	1.65 ± 0.17	0.19 ± 0.01	0.32 ± 0.02
		0.2	24.67 ± 0.4	17.1 ± 0.31	11.22 ± 0.2	0.93 ± 0.11	0.34 ± 0.03	11 ± 0.31	8.38 ± 0.27	13.52 ± 0.2	1.79 ± 0.10	0.17 ± 0.01	0.32 ± 0.04
	0.4	0	24.83 ± 0.3	16.61 ± 0.4	11.41 ± 0.5	0.52 ± 0.01	0.36 ± 0.01	10.65 ± 0.5	8.4 ± 0.31	13.4 ± 0.07	1.72 ± 0.38	0.2 ± 0.03	0.36 ± 0.03
		0.1	25.5 ± 0.22	17.16 ± 0.3	12.13 ± 0.2	1.25 ± 0.16	0.35 ± 0.01	11.37 ± 0.5	7.97 ± 0.44	13.3 ± 0.3	1.79 ± 0.15	0.19 ± 0.04	0.35 ± 0.02
		0.2	25 ± 0.52	17.91 ± 0.5	12.68 ± 0.2	1.62 ± 0.1	0.35 ± 0.02	10.63 ± 0.3	7.57 ± 0.27	13.37 ± 0.2	1.78 ± 0.33	0.2 ± 0.01	0.31 ± 0.03
10	0	0	33.33 ± 1.5	12.52 ± 0.3	8.19 ± 0.34	1.03 ± 0.09	0.38 ± 0.02	21.65 ± 0.9	20.33 ± 0.5	17.64 ± 0.9	2.9 ± 0.14	0.23 ± 0.03	0.84 ± 0.03
		0.1	34.17 ± 1.4	15.81 ± 0.3	8.83 ± 0.32	1.14 ± 0.09	0.37 ± 0.02	20.98 ± 0.3	19.42 ± 0.3	18.41 ± 0.2	3.12 ± 0.19	0.2 ± 0.01	0.86 ± 0.04
		0.2	34.17 ± 1.4	15.99 ± 0.6	7.57 ± 0.51	1.18 ± 0.07	0.36 ± 0.02	21.55 ± 0.3	17.5 ± 0.83	18.13 ± 0.1	3.08 ± 0.24	0.21 ± 0.02	0.88 ± 0.06
	0.1	0	29.5 ± 1.4	13.94 ± 0.6	10.49 ± 0.1	1.23 ± 0.08	0.41 ± 0.01	21.47 ± 0.5	18.73 ± 0.5	19.4 ± 0.3	3.2 ± 0.19	0.21 ± 0.02	0.93 ± 0.18
		0.1	35.83 ± 1.1	16.06 ± 0.3	11.28 ± 0.4	1.38 ± 0.07	0.43 ± 0.01	19.7 ± 0.74	17.3 ± 0.38	19.32 ± 0.5	3.03 ± 0.14	0.19 ± 0.03	0.93 ± 0.09
		0.2	35.33 ± 1.3	17.15 ± 0.3	11.3 ± 0.04	1.52 ± 0.09	0.44 ± 0	20.73 ± 1.0	19.4 ± 0.44	19.4 ± 0.08	3.11 ± 0.29	0.21 ± 0.03	1.04 ± 0.09
	0.2	0	35.17 ± 1.2	16.92 ± 0.4	13.07 ± 0.3	1.22 ± 0.07	0.44 ± 0.01	20.08 ± 0.3	16.35 ± 0.4	19.4 ± 0.06	3.14 ± 0.27	0.19 ± 0.01	1.07 ± 0.08
		0.1	38.5 ± 0.7	17.91 ± 0.7	14.2 ± 0.15	1.8 ± 0.1	0.44 ± 0	18.95 ± 0.4	15.23 ± 0.3	19.5 ± 0.1	3.23 ± 0.19	0.22 ± 0.04	1.06 ± 0.12
		0.2	32.67 ± 1.9	19.8 ± 0.5	15.33 ± 0.3	1.67 ± 0.08	0.46 ± 0.01	17.6 ± 0.38	15.03 ± 0.3	19.7 ± 0.09	3.19 ± 0.03	0.2 ± 0.01	1.05 ± 0.05
	0.4	0	35.17 ± 2.1	18.57 ± 0.3	13.95 ± 0.8	1.35 ± 0.14	0.45 ± 0	17.38 ± 0.3	15 ± 0.41	19.36 ± 0.07	3.56 ± 0.06	0.21 ± 0.02	1.08 ± 0.12
		0.1	36.17 ± 1.2	17.11 ± 0.3	17.13 ± 0.7	1.9 ± 0.17	0.45 ± 0.01	16.08 ± 0.5	14.1 ± 0.23	19.25 ± 0.2	3.68 ± 0.18	0.19 ± 0.03	1.15 ± 0.14
		0.2	37 ± 1.4	18.34 ± 0.6	18.6 ± 0.06	2.37 ± 0.11	0.47 ± 0.01	15.17 ± 0.8	14.17 ± 0.5	19.62 ± 0.1	3.78 ± 0.42	0.2 ± 0.02	1.17 ± 0.09
20	0	0	35.67 ± 1.3	12.26 ± 0.3	7.33 ± 0.16	2.8 ± 0.04	0.38 ± 0	32.8 ± 0.44	28.23 ± 0.7	18.27 ± 0.1	3.36 ± 0.38	0.25 ± 0.01	1.07 ± 0.17
		0.1	39.33 ± 1.3	12.32 ± 0.4	8.5 ± 0.22	3.28 ± 0.05	0.39 ± 0	31.95 ± 0.6	27.75 ± 0.4	17.49 ± 0.1	3.4 ± 0.22	0.23 ± 0.01	1.06 ± 0.09
		0.2	42.67 ± 1.4	11.96 ± 0.5	9.16 ± 0.31	3.7 ± 0.12	0.4 ± 0	31.48 ± 0.5	28.65 ± 0.7	18.41 ± 0.1	3.38 ± 0.18	0.23 ± 0.03	1.19 ± 0.14
	0.1	0	35 ± 1.06	13.76 ± 0.3	9.66 ± 0.35	2.92 ± 0.06	0.4 ± 0	31.23 ± 0.5	27 ± 0.36	19.23 ± 0.1	3.48 ± 0.26	0.24 ± 0.01	1.09 ± 0.11
		0.1	36.17 ± 1.2	13.95 ± 0.3	10.15 ± 0.3	3.18 ± 0.16	0.42 ± 0.01	30.92 ± 0.5	26.8 ± 0.39	20.54 ± 0.2	3.57 ± 0.42	0.22 ± 0.02	1.22 ± 0.35
		0.2	36.83 ± 1.1	16.67 ± 0.8	10.53 ± 0.3	3.9 ± 0.25	0.53 ± 0.01	31.53 ± 0.3	28.45 ± 0.5	20.34 ± 0.5	3.75 ± 0.34	0.22 ± 0.03	1.12 ± 0.23
	0.2	0	32.5 ± 2.4	14.53 ± 0.7	10.7 ± 0.04	2.57 ± 0.07	0.51 ± 0	31.83 ± 0.5	30.08 ± 0.6	20.2 ± 0.03	3.92 ± 0.21	0.22 ± 0.01	1.15 ± 0.19
		0.1	37.67 ± 1.6	17.13 ± 0.5	10.9 ± 0.55	3.53 ± 0.1	0.52 ± 0.01	29.77 ± 0.4	25.8 ± 0.26	20.75 ± 0.2	4.2 ± 0.12	0.22 ± 0.04	1.31 ± 0.15
		0.2	39.5 ± 2	18.05 ± 0.9	11.94 ± 0.	3.73 ± 0.15	0.6 ± 0	29.42 ± 0.4	25.57 ± 0.3	21.55 ± 0.4	4.37 ± 0.04	0.21 ± 0.01	1.29 ± 0.29
	0.4	0	37.67 ± 1.5	16.5 ± 0.6	12.64 ± 0.2	3.32 ± 0.1	0.54 ± 0	29.38 ± 0.3	25.43 ± 0.3	21.78 ± 0.3	4.47 ± 0.52	0.21 ± 0.03	1.34 ± 0.33
		0.1	38.83 ± 1.6	19.32 ± 0.2	13.46 ± 0.2	3.52 ± 0.08	0.62 ± 0.01	29.1 ± 0.36	25.13 ± 0.6	21.9 ± 0.04	4.62 ± 0.12	0.2 ± 0.01	1.33 ± 0.29
		0.2	35.67 ± 1.3	18.93 ± 0.3	13.36 ± 0.3	3.37 ± 0.13	0.64 ± 0	28.65 ± 0.5	23.35 ± 0.3	22 ± 0.06	5.16 ± 0.13	0.2 ± 0.02	1.53 ± 0.34
40	0	0	25 ± 1.1	8.39 ± 0.09	5.53 ± 0.38	2.15 ± 0.07	0.23 ± 0.01	38.28 ± 0.5	34.43 ± 0.5	16.88 ± 0.2	2.38 ± 0.06	0.27 ± 0.04	0.51 ± 0.26
		0.1	25.5 ± 1.1	8.16 ± 0.36	5.64 ± 0.17	2.08 ± 0.03	0.28 ± 0.01	34.87 ± 0.5	31.72 ± 0.3	17.31 ± 0.2	2.43 ± 0.30	0.24 ± 0.03	0.54 ± 0.17
		0.2	24.5 ± 0.8	8.2 ± 0.15	6.49 ± 0.21	2.52 ± 0.1	0.33 ± 0.01	32.25 ± 0.3	32.95 ± 1.6	16.85 ± 0.1	2.48 ± 0.15	0.25 ± 0.02	0.57 ± 0.18
	0.1	0	30.17 ± 1.6	8.67 ± 0.16	5.24 ± 0.04	2.05 ± 0.08	0.32 ± 0	32.27 ± 0.3	30.78 ± 0.5	16.85 ± 0.1	2.46 ± 0.17	0.24 ± 0.03	0.53 ± 0.3
		0.1	30 ± 1.18	9.22 ± 0.35	4.88 ± 0.34	2.85 ± 0.08	0.34 ± 0	32.32 ± 0.3	29.35 ± 1.5	17.24 ± 0.1	2.59 ± 0.35	0.24 ± 0.01	0.61 ± 0.21
		0.2	29.67 ± 0.9	8.72 ± 0.35	5.46 ± 0.06	2.65 ± 0.07	0.35 ± 0.01	31.78 ± 0.5	29.97 ± 0.4	17.67 ± 0.1	2.89 ± 0.44	0.23 ± 0.02	0.63 ± 0.24
	0.2	0	30.17 ± 1.5	9.01 ± 0.33	8.29 ± 0.33	2.32 ± 0.15	0.34 ± 0.01	32.12 ± 0.8	30.17 ± 0.4	17.13 ± 0.1	2.66 ± 0.18	0.24 ± 0.02	0.62 ± 0.15
		0.1	30.33 ± 1.1	10.51 ± 0.3	9.88 ± 0.32	2.5 ± 0.13	0.37 ± 0.01	31.18 ± 0.5	29.65 ± 0.3	17.11 ± 0.0	2.67 ± 0.11	0.22 ± 0.03	0.67 ± 0.07
		0.2	35.5 ± 0.99	10.91 ± 0.5	10.18 ± 0.3	3.12 ± 0.2	0.38 ± 0.01	29.82 ± 0.3	27.12 ± 1.8	17.35 ± 0.07	2.7 ± 0.17	0.23 ± 0.02	0.64 ± 0.14
	0.4	0	30.83 ± 1.3	10.48 ± 0.4	10.58 ± 0.3	2.32 ± 0.05	0.34 ± 0.01	30.55 ± 0.1	29.17 ± 0.4	17.03 ± 0.04	2.8 ± 0.23	0.22 ± 0.01	0.68 ± 0.07
		0.1	31.5 ± 1.2	11.98 ± 0.2	10.97 ± 0.3	2.7 ± 0.3	0.35 ± 0.01	28.35 ± 0.8	26.87 ± 1.4	17.56 ± 0.14	2.85 ± 0.16	0.23 ± 0.03	0.65 ± 0.07
		0.2	34 ± 1.4	14.08 ± 0.9	11.49 ± 0.3	2.47 ± 0.19	0.36 ± 0.00	28.43 ± 0.3	23.97 ± 2.6	17.43 ± 0.34	2.68 ± 0.09	0.21 ± 0.02	0.75 ± 0.27
LSD (0.05)			3.45	1.25	0.93	0.32	0.03	1.63	2.02	0.69	0.35	0.015	0.09

S: Salt (dS/m), P: SNP (Mm), K: KNO_3_ (%), PH: plant height, CB: chlorophyll b, CT: total chlorophyll, BY: biological yield, TW: thousand-grain weight, Na: concentration of Na+, Cl: concentration of Cl-, SO: Seed oil

**Fig. 2:**
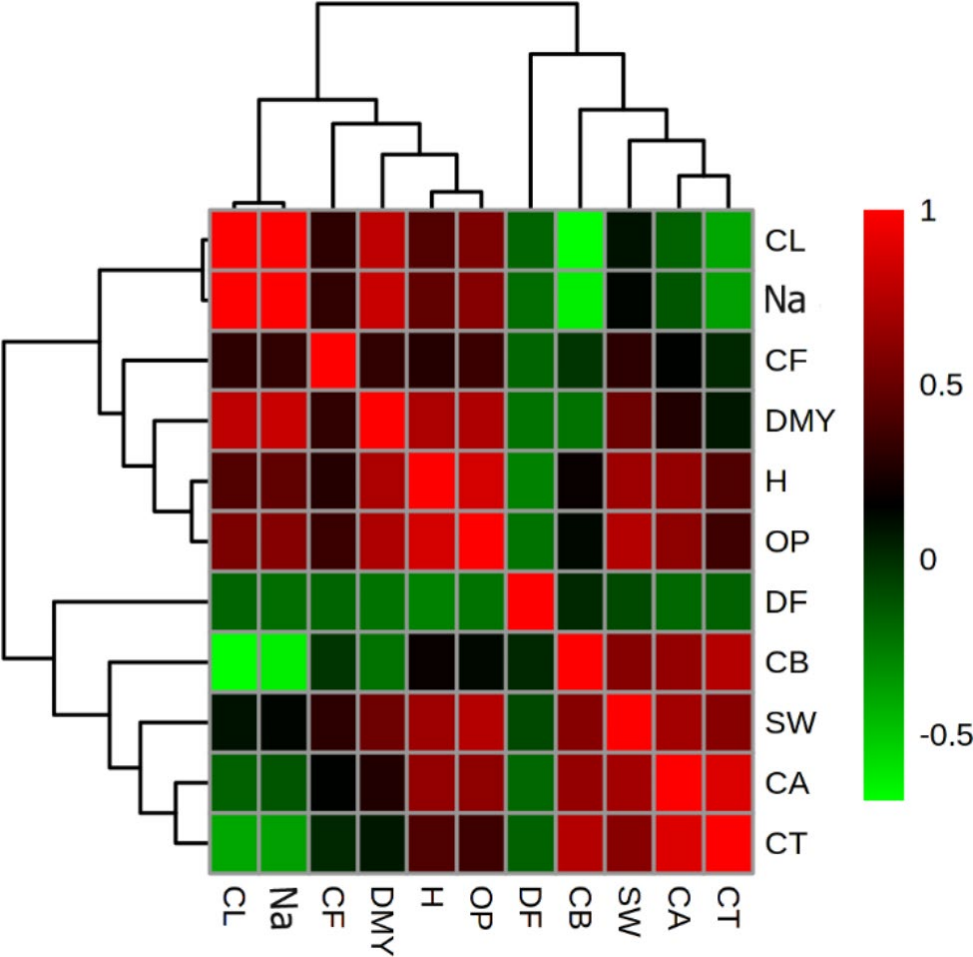
Heat map of the correlations between the characters studied under salt stress of *S. persica*. CT: total chlorophyll, CA: chlorophyll **a**, CB: chlorophyll **b**, SW: thousand-grain weight, DF: the number of day until flowering, OP: percentage of oil, H: plant height, DMY: biological yield, Na: concentration of Na^+^, Cl: concentration of Cl^−^

The lowest value of plant height (21 cm) occurred in response to 0 dS/m NaCl. These results showed that *S. persica* needed an optimum amount of NaCl in irrigation water for optimal growth (Table 3). Similar to the current results, Kong and Zheng [28] reported that the stem of plants grown on solutions of 6 to 10 mM NaCl was significantly shorter than those grown on 200 mM. Also, Katschnig et al. [29] showed that the growth rate of *Salicornia dolichostachya* was 123% greater when treated with 300 mM NaCl, compared to the control. Similarly, our results revealed that plant height had a positive significant correlation with the concentrations of Na^+^ and Cl^−^ (Fig. 2).

Rozema and Schat [30] demonstrated that when the availability of NaCl decreases, Na^+^ and Cl^−^ are stored in the cell wall instead of the vacuoles, leading to a decrease in turgor pressure. According to the results, the highest value of plant height (42.67 cm) was obtained by the application of potassium nitrate (1%) in the irrigation water, along with 20 dS/m NaCl.

It seems that using potassium nitrate along with NaCl improved the possibility of potassium absorbance, and alleviated the adverse effects of salt stress [31, 32]. Previous studies stated that nitrate potassium enhanced plant height and growth in different plants such as pistachio, melon, and wheat [32–34] under salt stress. The application of 0.2 mM SNP and 1% nitrate potassium prevented a severe decrease in plant height (35 cm) under the highest salinity level (40 dS/m of NaCl) in comparison with the control (25 cm). These results indicated that the applications of SNP and nitrate potassium enhanced the height of *S. persica* plants irrigated with 40 dS/m NaCl salt water.

### Chlorophyll content

The rate of photosynthesis depends on the amount of chlorophyll content in plants while chlorophyll degradation occurs during salt stress [35]. The results showed that 10 dS/m NaCl increased chlorophyll a, chlorophyll b, and total chlorophyll contents in comparison with the control. By increasing the amount of NaCl in irrigation water from 20 to 40 dS/m, a sharp decrease occurred in these contents. A previous study showed that using optimum concentrations of NaCl improved growth-related and yield traits of plants [28], indicating that the application of NaCl with suitable concentrations can lead to improvements in the chlorophyll content of *S. persica*.

Plants treated with SNP (0.4 mM) and KNO_3_ (0.1%) showed a 70–100% increase in chlorophyll a, chlorophyll b, and total chlorophyll contents under severe salinity stress. Gohari et al. [36] showed that the contents of chlorophyll a, chlorophyll b, and total chlorophyll content increased after SNP application during salt stress. Benedetti and Arrud [37] stated that reactive oxygen species (ROS) increased in chloroplasts during salt stress, which led to the degradation of chloroplasts. Our results showed that the application of SNP enhanced chlorophyll pigment formation, which was essential for improving the photosynthesis rate by protecting the membranes of cellular organelles containing chlorophyll. It is reportedly proven that the utilization of SNP-protected chlorophyll membranes involves increasing the activity of antioxidant enzymes that subsequently scavenge ROS [38–40]. Also, the diminished uptake of magnesium and potassium ions, the most important elements for chlorophyll production, occurred by an excessive presence of sodium ions, resulting in chlorophyll degradation. On the other hand, previous studies showed that chlorophyll content was affected by nitrogen and potassium and that using KNO_3_ increased leaf K^+^ and N concentrations, thereby increasing the chlorophyll content [41].

### Biological yield and thousand-grain weight

Biological yield is an important parameter for enhancing the yield of *S. persica* (especially as feed for animals), which is strongly affected by environmental factors [42]. In addition, the thousand-grain weight is the most important yield component that determines the final yield and is regarded as a potential selection criterion for yield. When salt stress is prolonged, it results in a decrease in photosynthetic rate and an increase in necrosis and chlorosis, resulting in reduced yield traits [38].

The exposure of *S. persica* plants to 0.1 or 2 mM of SNP and 1% of KNO_3_ under 20 dS/m NaCl led to an increase in biological yield (3.9 gr) and thousand-grain weight (0.64 gr). In the present study, the lowest amount of biological yield belonged to the control (without NaCl). Kong and Zheng (2014) demonstrated that the yields of *S. bigelovii* grown at low levels of salinity (5 mM NaCl) were significantly lower than those grown at high levels of salinity (200 mM NaCl). They showed that a decrease in the yield of *S. bigelovii* plants grown in 5 mM NaCl did not result from an insufficient supply of photoassimilates to support growth. The decrease of succulence (i.e. the amount of water per unit dry mass) could be a major contributor to reduced biological yield when this halophyte is grown at a moderate level of salinity [28]. Rozema and Schat (2013) reported that succulence could be a prerequisite for the salt-stimulated growth of halophyte plants. The results of the present study showed that increasing the concentration of salinity (to more than 20 dS/m) led to a decline in yield parameters, even though the parameters were enhanced by SNP and KNO_3_. Our results revealed that SNP enhanced chlorophyll pigment formation, which was essential for stimulating the photosynthesis rate by protecting cellular organelles that contain chlorophyll. Ultimately, this led to an increase in the photosynthesis rate under saline conditions. These results confirmed early findings by Gohari et al. [36] and Yasir et al. (2021) in *Ocimum basilicum* and *Lens culinaris*, respectively. The correlation analysis showed that the thousand-grain weight had a positive, significant correlation with chlorophyll a and total chlorophyll contents (Fig. 2). Our results indicated that the experimental treatments in the present study (KNO_3_ and SNP) strongly contributed to the increase in grain and biological yields by a moderate, salty irrigation (20 to 40 dS/m NaCl).

### Na^+^ and Cl^−^ ions

The triple interaction effects of NaCl, SNP, and KNO_3_ significantly affected the accumulation of Na^+^ and Cl^−^ ions in *S. persica*. The decrease in Na^+^ content is one of the most important survival strategies of plants during salt stress [43]. According to the results, concentrations of Na^+^ and Cl^−^ ions increased by about 2.5 and 3 times, compared to the control under salt stress. Excessive concentrations of Na^+^ and Cl^−^ ions in plants affected the uptake and metabolism of other nutritional elements [44]. Higher NaCl concentrations were toxic to plant cells and led to oxidative stress and damage to the cell membranes, especially chlorophyll [45]. Similarly, the obtained results showed that the concentration of Na^+^ and Cl^−^ negatively correlated with chlorophyll a, chlorophyll b, and total chlorophyll contents (Fig. 2). When plants are irrigated with water containing 40 dS/m NaCl, it caused the lowest concentrations of Na^+^ (28.35 mg g^− 1^ DW) and Cl^−^ (23.96 mg g^− 1^ DW) ions as a result of 0.2 mM SNP and 0.1% KNO_3_. These results showed that using SNP and KNO_3_ mitigated the adverse effects of salt stress through a decrease in Na^+^ and Cl^−^ accumulation in *S. persica*, thereby increasing the chlorophyll content. The application of SNP as NO donors revealed an amelioration effect on the measured traits by decreasing Na^+^ and Cl^−^ contents in sunflower plants during salinity stress [44]. An ionic balance was reportedly controlled by VH^+^-ATPase, PMH^+^-ATPase, and NO in reed [46] and *Arabidopsis* [47]. Kaya et al. (2003) showed that the applied concentration of supplementary KNO_3_ led to the mitigation of Na accumulation. Similarly, it was confirmed that Na^+^ is taken up by many K^+^ transporters and that a large amount of Na^+^ ions are aggregated in plant shoots by systems that show nitrate dependence. Thus, using mineral nutrients (specially KNO_3_) led to a reduction in Na^+^ accumulation.

### Seed oil and yield

The cultivation of *S. persica* under mixed treatments of NaCl, KNO_3_, and SNP affected the plant seed oil. The exposure of *S. persica* plants to salt stress resulted in a severe increase in the percentage of seed oil from 13.3 to 20.2%. Our results were in agreement with those observed by El-Araby et al. (2020), that increasing the salinity levels resulted in a gradual increase in oil content. The results of correlation analysis showed that the Na^+^ and Cl^−^ positively correlated with the percentage of oil. The highest seed oil (22.02%) was obtained in response to treatments of 0.4 mM SNP under 20 dS/m NaCl while the lowest values (13.17 to 13.51%) were observed in treatment groups without salt stress.

According to the results, the three salinity levels, including 10, 20, and 40 dS/m caused an increase in the seed oil percentage and the seed yield, compared to the 0 dS/m of NaCl (Table 3). These results indicated that the plant needs an optimal level of salinity (20 dS/m NaCl) to obtain more seed oil and yield [9]. The results also revealed that 40 dS/m NaCl led to a decrease in the seed oil percentage and seed yield, compared to the effect of 20 dS/m NaCl. This result showed that the highest level of salinity caused a decrease in plant oil and yield. By increasing the levels of the other two factors, i.e. SNP and KNO3, the seed oil percentage and seed yield increased. It was demonstrated that both SNP and KNO_3_ were useful in mitigating the adverse effects of high-level salinity on the oil and seed yield of plants.

The application of nitrogen increased photosynthesis, biomass, and oil yield in aromatic and medicinal plants [49]. Similarly, SNP is known to release nitrogen [36]. On the other hand, previous studies demonstrated that several endogenous signaling pathways regulated essential oil and that NO was identified as an essential signal molecule in elicitor-induced secondary metabolites [17, 50]. However, there is little information about the role of SNP on seed oil and seed yields in plants. The enhancement of seed yield and seed oil by foliar application of SNP may be related to the enhancement of nutrient uptake and cycle growth [50, 51]. The correlation analysis indicated that thousand-grain weight had a positive, significant correlation with the percentage of oil. Beyrami et al. [52] showed that as the salinity level of irrigation water increased from 8 to 25 dS.m-1, yield components significantly increased in *S. bigelovii* and *S. persica*. In the present study, 22% of the oil content was extracted from the harvested seeds. It seems that the oil content and seed yield are affected by different factors such as plant species, genotype, culture, and climatic conditions.

### Number of days to flowering

NaCl, SNP, and KNO_3_ significantly affected the number of days to flowering (NDF). The increase of NaCl concentration in irrigation water accelerated the onset of reproductive growth and led to the decrease of NDF from 195 to 168 days, while the application of SNP and KNO_3_ enhanced the amount of this trait. The results of correlation coefficients showed that the NDF had a negative, significant correlation with concentrations of Na^+^ and Cl^−^. The application of nitrogen sources delayed generative growth and increased the NDF or vegetative growth in different plants [53]. Seligman et al. [54] showed that the utilization of SNP exogenously delayed flowering in *Arabidopsis* plants. It was confirmed that SNP as a NO donor repressed the expression of genes dependent on the circadian clock and an increase in the accumulation of mRNA that encodes a key repressor of flowering (FLC) [55]. In addition, Zhang et al. [56] showed that nitrogen and NO postpone plant flowering by regulating several genes such as ferredoxin NADP + oxidoreductase (FNR1), the blue-light receptor cryptochrome 1 (CRY1), CO (CONSTANS), and GI (GIGANTEA).

### Antioxidant enzymes

**Fig. 3:**
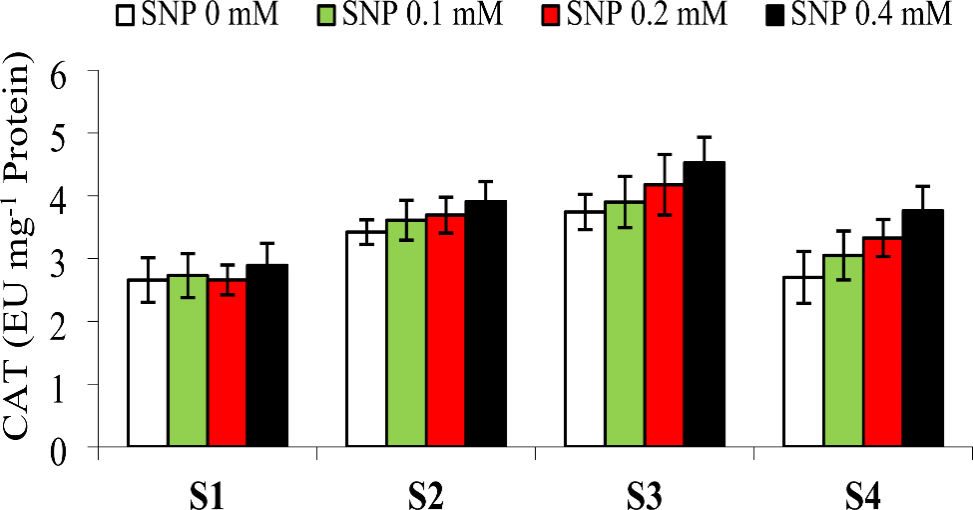
Effects of application of NaCl (S1 to S4: 0, 10, 20 and 40 ds/m, respectively) and SNP (0, 0.1, 0.2 and 0.4 mM) on activity of CAT enzyme in *S. persica* plants. Different letters indicate significant differences among means using LSD test at P = 0.05

The activity of antioxidant enzymes can be one of the important indicators that help to determine the resistance of plants to salt stress conditions. The results observed for SOD and CAT enzyme activity showed that these enzymes, under control conditions, had suppressed activities, compared to the other treatments. Increasing the SNP concentration had a significant effect (P < 0.01) on the activity of antioxidant enzymes and led to the simulation of SOD and CAT. We found that foliar spraying with KNO_3_ had the maximum positive effect on SOD and CAT enzyme activity. Also, our results showed that the activity of SOD and CAT enzymes increased under salt stress in comparison with the control treatment. The results revealed that by increasing the SNP concentration, the antioxidant enzymes SOD and CAT increased. Also, the results of double interaction between SNP and NaCl significantly revealed that the highest and lowest activities of CAT were observed in response to the foliar spraying of 0.4 mM SNP + 20 ds/m NaCl and control (0 mM SNP + 0 ds/m NaCl) treatments (Fig. 3).

The results of triple interaction among the factors (KNO_3_× SNP× NaCl) showed a significant difference in the SOD enzyme. These results demonstrated that the highest amount of SOD (5.16 EU mg^− 1^ protein) was found in 2% KNO_3_ with 0.4 mM SNP under 20 dS/m NaCl.

The inhibition of redox status disturbances and oxidation of cellular components caused by stress-derived ROS are functions of enzymatic and non-enzymatic antioxidants for the survival of plants undergoing stress conditions [57]. In this study, we observed that the trend of increasing enzyme activity directly correlated with elevated levels of NaCl from 0 to 20 ds/m, but then dropped rapidly in response to severe salt stress. Although CAT and SOD activities increased considerably in the NaCl treatments, they did not reach the high levels of SNP treatments. It was reported that the increase of NO and potassium-stimulated stress tolerance occurred through the activation of antioxidant enzymes and resulted in the alleviation of ROSs concentration in stressed plants [38]. Similarly, the results showed that the activity of CAT and SOD enzymes increased linearly when the amounts of SNP and KNO_3_ were increased. These results provided support for previous reports documenting the enhanced activity of antioxidant enzymes by the application of KNO_3_ and SNP [57, 58].

### Malondialdehyde (MDA)

The results of simple effects showed that the highest MDA was found in the severe salinity-stress treatment (40 dS/m of NaCl), while the lowest MDA was recorded in the control. The present results indicated that by increasing the concentrations of both factors, i.e. SNP and KNO_3,_ the MDA content decreased. Also. the results indicated that the foliar spray with SNP + KNO_3_ ameliorated the MDA parameter in *S. persica* under severe salinity stress.

The foliar spray with either KNO_3_ or SNP and subsequent exposure to NaCl resulted in the mitigation of lipid peroxidation and the preservation of membrane integrity, as showed by lower levels of MDA contents in comparison with NaCl-alone exposed plants. Lower levels of lipid peroxidation after treatment with NaCl in response to either KNO_3_ or SNP pretreatments have also been reported in different plants such as lentils [38], strawberries [57], and wheat [59]. We found that the increase in SNP concentration led to a decrease in MDA and that these indexes showed the lowest corresponding values, where the maximum dose of SNP (0.4 mM) was added to the improvement of adverse effects of severe salt stress in *S. persica*. It was reported that 152 genes from 214 sequenced transcript fragments in *Nicotiana tabacum* were independently induced by nitric oxide, showing a vigorous overlapping function in the signaling pathways triggered by nitric oxide [60]. Also, Qiao and Fan, [61] reported that signaling networks between NO and H_2_O_2_ can serve as important factors for the regulation of plant responses to imbalances in metabolic activities. Kaya and Higgs [48] reported that the application of KNO_3_ led to the mitigation of Na^+^ ions which otherwise accumulated and resulted in a decrease in MDA and electrolyte leakage. These reports showed that KNO_3_ and NO improved membrane stability by controlling ionic balance under salt stress [47].

### Proline content

Salinity enhanced proline content compared to the control. Maximum proline content (0.27 mg/g FW) was observed in response to the severe salinity stress without the application of KNO_3_ or SNP. Furthermore, the lowest values of proline content belonged to non-stressed plants. Some treatments inhibited the increase of proline content under severe salinity stress as compared with the control condition, such as 0.2 mM SNP + 1% KNO_3_, and 0.4 mM SNP + 1% KNO_3_ treatments, suggesting that they mitigated proline induction under salt stress in comparison with control conditions. Plants activate their defensive system by overproducing proline and preserving the inner water content of the cells as their self-defense mechanism under salt stress [62]. Our results showed that exogenous KNO_3_ and SNP promoted salt stress tolerance through the regulation of proline content. The increase and accumulation of proline as an osmoprotective agent and a radical scavenger in cells is a primary response of plants to abiotic stresses and protects cells against damage [63]. The accumulation of proline in salt-stressed plants is in line with earlier results concerning *S. bigelovii* [64], *S. europaea* [65], and *S. persica* [66]. Proline is a compatible solute that protects different sensitive enzymes against sodium ions in the cytoplasm.

## Conclusions

This study suggested that a moderate application of NaCl (20 dS/m) combined with proper levels of SNP (0.2 mM) and KNO_3_ (0.1%) at the establishment stage of *S. persica* plants can be a promising approach to obtain higher seed yield and seed oil. Also, the application of 0.4 mM SNP and 0.2% KNO_3_ could mitigate the adverse effects of severe salt stress (40 dS/m) on plant characteristics. The applications of SNP and KNO3 at farm level can lead to better growth, yield, and production of halophytes plants under saline conditions, thereby securing future food production.

## Data Availability

All data are within the manuscript.
